# Mindfulness, Self-Efficacy, Job Stress, and Job Satisfaction in Associated Factors of Turnover Intention: A Regression-Based Path Analysis Among Direct Care Workers

**DOI:** 10.3390/healthcare14050654

**Published:** 2026-03-04

**Authors:** Hsuan-Pin Chen, Kuo-Chung Huang

**Affiliations:** Department of Business Administration, Nanhua University, Chiayi 62249, Taiwan; kchuang@nhu.edu.tw

**Keywords:** mindfulness, self-efficacy, job stress, job satisfaction, turnover intention, direct care workers

## Abstract

**Background/Objectives:** This study aimed to examine how mindfulness and self-efficacy are associated with turnover intention among direct care workers through the hypothesized indirect pathways involving job stress and job satisfaction. Grounded in the Job Demands–Resources (JD–R) and Conservation of Resources (COR) frameworks, the study highlights the buffering and protective functions of psychological resources under high job demands. **Methods:** A regression-based path analysis was conducted using data collected from a structured questionnaire survey of 967 direct care workers in southern Taiwan. **Results:** Job stress was positively associated with turnover intention (β = 0.599, *p* < 0.001), whereas job satisfaction was negatively associated with it (β = −0.139, *p* < 0.001). Self-efficacy was positively associated with job satisfaction (β = 0.407, *p* < 0.001) and negatively associated with job stress (β = −0.109, *p* < 0.001). Mindfulness demonstrated significant direct associations with self-efficacy (β = 0.497, *p* < 0.001) and job stress (β = −0.200, *p* < 0.001), but its direct effect on turnover intention was not significant (β = −0.035, *p* > 0.05), implying its influence is indirect through self-efficacy, job stress, and job satisfaction. Diagnostic checks, including the Variance Inflation Factor (VIF), confirmed the absence of multicollinearity issues, and the overall model demonstrated satisfactory explanatory power. **Conclusions:** These findings enhance understanding of the psychological mechanisms underlying turnover intention among care workers and provide practical implications for human resource management and workplace stress interventions in long-term care settings.

## 1. Introduction

With advancements in medical technology and extended life expectancy, global societies are rapidly aging. Taiwan officially entered the “super-aged society” defined by the World Health Organization in 2025, with the proportion of adults aged 65 and above projected to rise from 19.2% in 2024 to 46.5% by 2070 [1]. This demographic shift has generated an urgent demand for long-term care while simultaneously creating a shortage of care workers. Direct care workers, who are the frontline personnel providing hands-on assistance in the long-term care system, face high turnover rates that threaten the sustainability and quality of care services [2,3].

Job stress and emotional labor are major contributors to turnover intention among care workers [3,4,5]. When job demands consistently exceed individual resources, burnout and reduced job satisfaction occur, leading to higher turnover intention [3,6,7,8]. Conversely, job satisfaction has been identified as a critical protective factor against turnover intention [9,10,11]. Enhancing psychological resources and resilience has therefore become an essential strategy in sustaining the care workforce.

Mindfulness, defined as conscious awareness and nonjudgmental attention to the present moment [12,13], is regarded as a personal resource that promotes self-efficacy, emotional regulation, and well-being while reducing stress [14,15,16]. In workplace research, mindfulness has been found to reduce turnover intention indirectly through its positive associations with self-efficacy, job stress, and job satisfaction [8,13,17]. However, empirical research focusing on mindfulness among direct care workers remains limited. This specific population faces unique pressures, including prolonged working hours, relatively lower wages, and high occupational risks [3,18], which distinctively influence their psychological resource utilization and job retention compared to other healthcare professionals.

This study is grounded in the Job Demands–Resources (JD–R) and Conservation of Resources (COR) frameworks. According to JD-R theory [19,20], the balance between job demands and resources determines employees’ stress and well-being. COR theory posits that individuals strive to conserve and accumulate psychological resources to manage stress [21]. Within these frameworks, mindfulness and self-efficacy are conceptualized as core personal resources that buffer the effects of high job demands and enhance job satisfaction [22,23]. Drawing on the JD-R and COR frameworks, this study uniquely tests a comprehensive path model investigating the indirect effects of mindfulness and self-efficacy on turnover intention, mediated by job stress and job satisfaction, specifically within the under-researched direct care worker population, thereby offering timely theoretical and practical implications for workforce management in long-term care settings.

Mindfulness (MF), defined as intentional and nonjudgmental awareness of present-moment experiences, is regarded as a core personal resource consistent with the Job Demands–Resources (JD–R) and Conservation of Resources (COR) frameworks. Empirical findings indicate that MF can significantly reduce stress, burnout, and psychological distress while enhancing work engagement, well-being, and job satisfaction [14,15,16]. MF also is linked to lower emotional exhaustion, strengthens emotional regulation and stability [13,24], and enhances resilience among healthcare and long-term care workers [15,24]. These effects suggest that MF may mitigate the adverse consequences of job stress on turnover intention.

Mechanism of Mindfulness against Psychological Inflexibility and Emotional Dysregulation: The core feature of Mindfulness (MF), which is non-judgmental awareness, stands directly opposed to the concept of Psychological Inflexibility. In high-pressure caring contexts (such as during the COVID-19 Pandemic), psychological inflexibility has been shown to be a crucial mediator between emotional dysregulation and occupational stress [25]. MF functions by enhancing psychological flexibility and emotional regulation abilities, enabling care workers to cope more adaptively with high demands, thereby preventing stress from escalating into turnover intention [14,15].

Overall, MF operates as a key personal resource that enhances self-efficacy (SE) and job satisfaction (JSA) while reducing job stress (JS), thereby potentially lowering turnover intention. Accordingly, this study hypothesizes that MF is positively associated with SE (H1) and JSA (H3), and negatively associated with JS (H2) and turnover intention (H4).

Self-efficacy refers to an individual’s belief in their ability to execute specific actions required to achieve desired goals through interactions with the physical and social environment [17,26,27]. According to the Conservation of Resources (COR) theory, self-efficacy constitutes a core psychological resource that enables individuals to mobilize energy and maintain stability when facing stress and challenges [21]. Similarly, the Job Demands–Resources (JD–R) theory posits that such personal resources buffer the detrimental effects of excessive job demands while enhancing job satisfaction and performance [19].

In the nursing and caregiving context, self-efficacy is recognized as a critical factor that promotes positive caregiving behaviors, enhances performance, and strengthens occupational satisfaction [17,27,28]. Empirical studies show that care workers and nurses with higher self-efficacy exhibit better emotional regulation, problem-solving abilities, and adaptability in high-pressure work situations [7,27,28,29]. High self-efficacy is also associated with lower burnout and a stronger willingness to remain in caregiving roles [26,29,30]. Furthermore, self-efficacy has been positively associated with job satisfaction and negatively associated with turnover intention [26,28,29].

Integration of High-Pressure Clinical Settings Literature: The robustness of the COR and JD–R frameworks has been validated across various high-pressure clinical environments. For instance, systematic reviews on ICU nurse burnout indicate that stress-related outcomes follow complex multilevel pathways [31], closely resembling the resource-driven sequential model proposed in this study. These findings strongly indicate that when faced with high-risk, high-demand caregiving roles (such as in intensive care units or emergency medicine), personal resources like Self-Efficacy and Mindfulness are critical for preventing emotional exhaustion and sustaining work engagement. This integration serves to reinforce the generalizability and theoretical robustness of our hypotheses.

In high-stress and emotionally demanding caregiving environments, fostering self-efficacy is critical to enhancing well-being, stabilizing workforce retention, and improving care quality [17,27,29]. In summary, self-efficacy is considered a key psychological resource that influences job satisfaction and turnover intention among care workers. This study hypothesizes that self-efficacy is positively associated with job satisfaction (H6) and negatively associated with turnover intention (H7).

Job Stress (JS), the core element of the health-impairment process within the JD–R framework, is commonly defined as the psychological, physiological, and emotional response that arises when job demands exceed employees’ available resources or perceived control [32,33]. According to the literature, unmanaged job stress is linked to lower job satisfaction [2,5,17,31] and undermines organizational stability [32,33]. Job stress is not only positively associated with turnover intention but also indirectly influences it through decreased job satisfaction [3,6]. Furthermore, job stress is negatively associated with self-efficacy, suggesting that persistent strain may weaken employees’ psychological resources [3].

In caregiving occupations, job stress arises from emotional demands, heavy workload, role conflict, time pressure, and anxiety pressure [3,18]. These diverse stressors form a unified high-order Job Demand construct, consistent with the JD–R and COR frameworks, which posit that stress emerges when individuals fail to obtain or maintain adequate resources [19,21].

Overall, job stress is a key variable associated with care workers’ well-being and turnover intention. This study hypothesizes that job stress is negatively associated with self-efficacy (H5), negatively associated with job satisfaction (H8), and positively associated with turnover intention (H9) [3,6].

Job satisfaction (JSA) has long been regarded as a central construct in organizational behavior, reflecting an individual’s overall evaluation of work experiences and their affective response to the job. It is defined as a pleasurable emotional state resulting from the appraisal of one’s job experiences [34,35]. Recent studies emphasize its multidimensional nature, influenced by work design, environment, interpersonal relationships, promotion opportunities, and supervisor support [36,37,38]. According to the Job Demands–Resources (JD–R) theory, JSA functions as an outcome of effective resource utilization. When sufficient resources—such as supervisory support, self-efficacy, and training opportunities—are available to balance high job demands, employees experience greater satisfaction and well-being [11,39]. Conversely, prolonged exposure to stress or insufficient resources is linked to lower JSA and increases turnover intention [8,37].

In the caregiving context, lower satisfaction levels lead to burnout, absenteeism, and higher turnover intention [2,4]. Empirical findings indicate that JSA mediates the relationship between job stress and turnover intention, reducing the positive association between stress and turnover [40,41]. Organizational factors such as supervisor support and training opportunities are crucial resources that enhance satisfaction and reduce turnover. Thus, enhancing JSA is critical for improving employees’ psychological well-being, promoting retention, and ensuring care quality. This study hypothesizes that job stress is negatively associated with JSA (H8), and JSA is negatively associated with turnover intention (H10).

Turnover intention (TI), defined as the psychological tendency or intent to leave one’s current job, remains a central issue in human resource management, directly influencing performance and organizational sustainability. High employee turnover not only increases recruitment and training costs but also undermines organizational continuity and overall productivity [37]. In the long-term care sector, high turnover rates threaten the sustainability and quality of care services, particularly as the demand for direct care workers rises rapidly in super-aged societies like Taiwan [2,3].

From the perspective of the Job Demands–Resources (JD–R) theory, TI is viewed as the ultimate outcome of resource depletion or imbalance [19]. When care workers face excessive job demands without sufficient personal or organizational resources, they experience stress and reduced job satisfaction, which eventually lead to turnover intention [11,39]. Conversely, job satisfaction has been widely recognized as a major negative predictor of turnover intention [34,35,38].

### 1.1. Research Framework and Hypotheses

**Figure 1:**
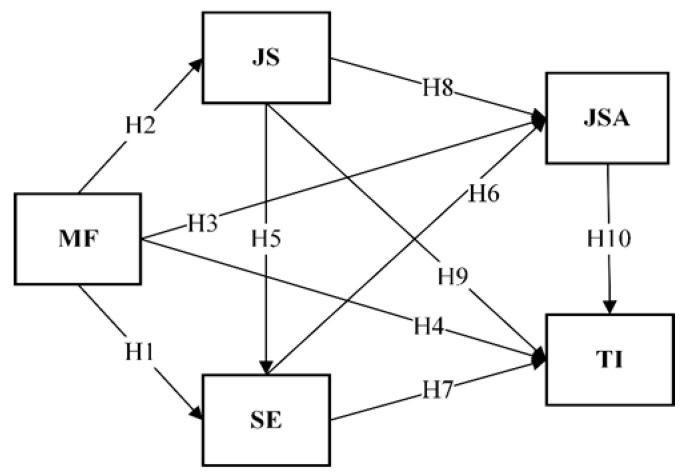
The proposed research framework of the study.

Therefore, this study is grounded in the JD-R and COR frameworks to investigate a comprehensive path model (Figure 1) aiming to clarify the resource-driven mechanisms through which psychological resources (Mindfulness and Self-Efficacy) and intervening variables (Job Stress and Job Satisfaction) influence turnover intention among direct care workers.

### 1.2. Drawing from the Theoretical Reasoning and Literature Review, the Following Hypotheses Were Proposed

**H1:**  *MF is positively associated with SE.*

**H2:**  *MF is negatively associated with JS.*

**H3:**  *MF is positively associated with JSA.*

**H4:**  *MF is negatively associated with TI.*

**H5:**  *JS is negatively associated with SE.*

**H6:**  *SE is positively associated with JSA.*

**H7:**  *SE is negatively associated with TI.*

**H8:**  *JS is negatively associated with JSA.*

**H9:**  *JS is positively associated with TI.*

**H10:**  *JSA is negatively associated with TI.*

**H11:**  *Mindfulness is hypothesized to have an indirect effect on turnover intention, mediated by job stress and job satisfaction*.

**H12:**  *Self-efficacy is hypothesized to have an indirect effect on turnover intention, mediated by job satisfaction.*

## 2. Materials and Methods

### 2.1. Study Design and Participants

This study adopted a cross-sectional research design and adopted a convenience sampling approach using anonymous paper-based questionnaires. The participants were direct care workers employed in long-term care institutions located in Kaohsiung City and Pingtung County, southern Taiwan, including nursing homes, adult day-care centers, and home-care service units.

Eligible participants were required to be at least 20 years old, hold a valid caregiving qualification, and have at least six months of work experience. Administrative personnel and temporary employees were excluded from the analysis. A total of 1050 questionnaires were distributed between 15 September and 10 December 2024, and 967 valid responses were collected, resulting in a valid response rate of 92.1%. The questionnaires were distributed through institutional coordinators, and participants completed them anonymously during working hours before submission. Although the sample provided important empirical evidence from frontline care workers in long-term care institutions in southern Taiwan, the use of a convenience sampling method may limit the generalizability and external validity of the findings. Future research could expand the sampling scope to enhance representativeness.

This study followed ethical research principles. All data were collected anonymously, without identifiable personal information or any intervention. Participation was voluntary, and participants completed the questionnaire after being informed of the study objectives.

### 2.2. Power Analysis and Sample Size Justification

To ensure that the sample size provided sufficient statistical power, the required sample size was estimated based on the guidelines proposed by Cohen [42] (pp. 109–114). A medium effect size (f^2^ = 0.15), a significance level of α = 0.05, and a statistical power of 0.80 were adopted for the estimation. Under these conditions, the minimum required sample size for a multiple regression model with up to six predictors was approximately 98 participants. The choice of a medium effect size was based on Cohen’s recommendations for behavioral science research, which aligns with the design context of this study. The actual valid sample size of 967 participants far exceeded this threshold, indicating that the study had sufficient statistical power to support the robustness and reliability of subsequent path regression analyses.

### 2.3. Research Framework

Based on the literature review, relationships exist among mindfulness (MF), self-efficacy (SE), job stress (JS), job satisfaction (JSA), and turnover intention (TI) among direct care workers. To verify these relationships, the research framework illustrated in Figure 1 was established. The framework posits that mindfulness and self-efficacy are expected to reduce job stress and enhance job satisfaction; job stress is positively associated with turnover intention, whereas job satisfaction negatively predicts it. These direct and indirect relationships are formally specified in hypotheses H1–H12.

### 2.4. Research Instruments

This study employed a structured questionnaire consisting of two main sections. The first section gathered demographic information of the respondents, including gender, marital status, nationality, age, level of education, total years of experience as a caregiver, the number of clients cared for per week, and monthly income. The second section focused on the core research variables and included five constructs: MF, JS, SE, JSA, and TI. Measurement Instruments: Five validated instruments were used to measure the five core constructs of this study: MF, JS, SE, JSA, and TI. The number of items for each construct was as follows: MF = 16 items, SE = 12 items, JS = 15 items, JSA = 13 items, and TI = 6 items. All instruments were selected based on their theoretical grounding and widely demonstrated strong psychometric properties in prior occupational and healthcare research. In line with this, our aggregation approach was theory-driven, guided by the JD–R and COR conceptualization of broad resource and demand categories rather than narrow facet-level modeling. Unless otherwise noted, all items were rated on a 7-point Likert scale ranging from 1 (*strongly disagree*) to 7 (*strongly agree*), with higher scores indicating stronger levels of the construct.

The Chinese versions were developed through a translation and back-translation procedure and reviewed by bilingual experts in occupational psychology and geriatric care to ensure semantic and cultural equivalence.

Notably, all instruments used in this study adopted previously validated Chinese versions that had undergone formal translation, cultural adaptation, and psychometric validation for Taiwanese occupational populations. Reliability and validity results are presented in Table 1 (Cronbach’s α = 0.775–0.958; KMO > 0.86; Bartlett’s *p* < 0.001).

#### 2.4.1. Mindfulness

MF was conceptualized as a trait-based psychological disposition, reflecting individuals’ awareness and attention to present-moment experiences in workplace contexts. Measurement was conducted using three widely validated scales: The Mindful Attention Awareness Scale (MAAS) developed by Brown and Ryan [43], assessing dispositional mindfulness. The Five-Facet Mindfulness Questionnaire (FFMQ) by Baer et al. [44], capturing five key dimensions: observing, describing, acting with awareness, non-judging, and non-reactivity. The Work Mindfulness Scale developed by Zivnuska, Kacmar, Ferguson, and Carlson [45], measuring mindfulness specifically in occupational settings. These instruments collectively capture both general and work-specific mindfulness traits relevant to caregiving contexts. In this study, the aggregation of general and work-specific scales was a deliberate methodological choice. Rather than merging incompatible constructs, this approach aims to capture complementary manifestations of the same underlying phenomenon—namely, mindful awareness across both general and occupational contexts. This conceptualizes Mindfulness as a holistic, higher-order personal resource, consistent with the resource-driven mechanisms of the JD-R and COR frameworks.

#### 2.4.2. Job Stress

JS was assessed using two classic and widely used scales. The JS Questionnaire (JSQ) developed by Caplan and Cobb [46] evaluates multiple dimensions of occupational stress including emotional, workload, and role stress. Additionally, the Job Stress Scale developed by Parker and De Cotiis [47] measures two major stress dimensions: time pressure (5 items) and anxiety pressure (4 items). These instruments provide a comprehensive assessment of stressors commonly experienced in caregiving occupations. The combination of these instruments aims to capture complementary manifestations of overall exposure to job demands across multiple stress domains (emotional, workload, role conflict, and time pressure), generating a unified high-order Job Demand construct necessary for modeling the overall health impairment process.

#### 2.4.3. Self-Efficacy

SE was measured using the General Self-Efficacy Scale (GSE) by Zhang and Schwarzer [48], which assesses individuals’ perceived capability to manage challenging situations. This construct was further grounded in Bandura’s [49] self-efficacy theory and Riggs et al.’s [50] organizational self-efficacy framework. Items capture caregivers’ confidence in handling job responsibilities, solving work-related problems, and achieving performance goals.

#### 2.4.4. Job Satisfaction

JSA was measured using the Minnesota Satisfaction Questionnaire (MSQ) developed by Weiss, Dawis, England, and Lofquist [51], which evaluates both intrinsic satisfaction (e.g., autonomy, sense of achievement) and extrinsic satisfaction (e.g., pay, working conditions). Additional items from Babin and Boles [52] and Cammann, Fichman, Jenkins, and Klesh [53] were included to capture organizational support, interpersonal relations, and promotion opportunities, providing a multidimensional view of job satisfaction.

#### 2.4.5. Turnover Intention

TI, defined as the psychological tendency or intent to leave one’s current job, was measured using validated items adapted from Scott et al. [54], Jung, Namkung, and Yoon [55], Kelloway, Gottlieb, and Barham [56], Mobley [57], and Michaels and Spector [58]. Representative items include “I often think about quitting my job” and “I am actively seeking another position”. All items were measured using a 7-point Likert scale, with higher scores indicating stronger turnover intention.

#### 2.4.6. Construct Validity and Reliability

All instruments demonstrated strong psychometric properties and have been validated extensively in prior occupational and organizational studies. Content validity was ensured by adopting items from theoretically grounded and widely cited instruments. Internal consistency reliability was excellent, with Cronbach’s α values ranging from 0.85 to 0.96, indicating that all scales possessed robust reliability and validity for measuring the intended constructs. Common Method Bias (CMB) was assessed using Harman’s single-factor test, with the largest single factor accounting for less than 40% of the variance, suggesting that CMB was not a significant concern.

### 2.5. Data Analysis Methods

Data entry and analysis were conducted using IBM SPSS Statistics for Windows, Version 22.0 (IBM Corp., Armonk, NY, USA) The level of statistical significance was set at *p* < 0.05. Group differences in demographic characteristics were analyzed using independent sample *t*-test and one-way analysis of variance (ANOVA). For variables that reached statistical significance in ANOVA, post hoc tests were conducted using either the Scheffé method or Dunnett’s T3 test, depending on variance homogeneity. Relationships among research variables were examined using linear regression analysis and path regression modeling. Data Screening and Assumption Testing: Prior to hypothesis testing, data were thoroughly screened. Due to a high valid response rate (92.1%), minimal missing data were addressed using pairwise deletion. Outliers were identified using standardized Z-scores (with scores exceeding ±3.29 flagged as extreme); however, upon inspection, extreme values were retained as they represented legitimate high/low responses, ensuring the integrity of the original dataset. Furthermore, we assessed the assumptions underlying Ordinary Least Squares (OLS) regression. The results confirmed that the assumptions of normality, linearity, and homoscedasticity were adequately met (e.g., visual inspection of residual plots and variance tests). VIF diagnostics (Max VIF = 1.626) confirmed the absence of multicollinearity.

## 3. Results and Analysis

### 3.1. Reliability and Validity Analysis

According to the results shown in Table 1, the Cronbach’s α values for all constructs exceeded the standard threshold of 0.7, indicating high internal consistency and strong correlations among the variables measured by the instrument. The suitability of the data for factor analysis was assessed using the Kaiser-Meyer-Olkin (KMO) measure and Bartlett’s test of sphericity. The analysis results for each construct are as follows: For MF, the KMO value is 0.864, and Bartlett’s test of sphericity is significant (*p* < 0.001), with a cumulative explained variance of 62.650%. The factor loadings range from 0.649 to 0.867. For JS, the KMO value is 0.941, and Bartlett’s test is significant (*p* < 0.001), with a cumulative explained variance of 71.118%. Factor loadings range from 0.705 to 0.881. For SE, the KMO value is 0.863, and Bartlett’s test is significant (*p* < 0.001), with a cumulative explained variance of 61.143%. Factor loadings range from 0.536 to 0.868. For JSA, the KMO value is 0.927, and Bartlett’s test is significant (*p* < 0.001), with a cumulative explained variance of 66.245%. Factor loadings range from 0.676 to 0.889. For TI, the KMO value is 0.952, and Bartlett’s test is significant (*p* < 0.001), with a cumulative explained variance of 69.830%. Factor loadings range from 0.580 to 0.891.

These EFA results-including high KMO values (MF: 0.864; JS: 0.941), strong factor loadings (all > 0.60), and the high cumulative variance explained by a single factor (MF: 62.650%; JS: 71.118%)-provide strong empirical support for treating the aggregated scales for Mindfulness and Job Stress as robust, unidimensional, high-order constructs suitable for the current path analysis model. These empirical results confirm that the aggregated scales effectively represent the same underlying phenomenon across different theoretical tools, supporting their use as robust, unidimensional, high-order constructs.

### 3.2. Sample Demographic Profile

Among the 967 valid responses, females accounted for the majority of the sample (84.7%, *n* = 819). In terms of marital status, 55.7% (*n* = 539) were married. Taiwanese nationals constituted 89.8% of respondents (*n* = 868). The most represented age group was 50–59 years (32.2%, *n* = 311), and the highest educational level reported was high school (47.3%, *n* = 457). Regarding work experience, 27.1% (*n* = 262) had been caregivers for more than one year but less than or equal to three years. In terms of workload, 40.8% (*n* = 395) cared for 5 to 6 clients per week. For monthly income, 48.0% (*n* = 464) reported earning between NTD 20,000 and 35,000. Descriptive statistics for each variable are presented in Table 2.

### 3.3. Regression Analysis

This study conducted a series of regression analyses to examine the association structure among MF, JS, SE, JSA, and TI. The results are summarized in Table 3:

1. Association between MF and JS

MF was significantly negatively associated with JS (β = −0.200, *p* < 0.001). The model explained 4.0% of the variance (R^2^ = 0.039, F = 52.595), indicating that higher mindfulness levels were related to lower job stress.

2. Association between MF and SE

MF was significantly positively associated with SE (β = 0.518, *p* < 0.001). The model explained 26.9% of the variance (R^2^ = 0.269, F = 354.722), indicating that higher mindfulness was related to increased self-efficacy.

3. Association between MF and JSA

MF was significantly positively associated with JSA (β = 0.352, *p* < 0.001). The model explained 12.4% of the variance (R^2^ = 0.124, F = 136.813).

4. Association between MF and TI

MF was significantly negatively associated with TI (β = −0.225, *p* < 0.001). The model explained 5.1% of the variance (R^2^ = 0.051, F = 51.391).

5. Association between JS and SE

JS was significantly negatively associated with SE (β = −0.209, *p* < 0.001). The model explained 4.4% of the variance (R^2^ = 0.044, F = 44.209).

6. Association between JS and JSA

The association between job stress and job satisfaction reached a significant level (β = 0.107, *p* < 0.001). The model explained a variance of R^2^ = 0.010, with an F value of 11.222. This may reflect that care workers are frequently required by care recipients to respond to various situations or handle complex service tasks. They may view the process of solving challenging work demands as a source of achievement, thereby experiencing satisfaction even in high-pressure environments.

7. Association between JS and TI

JS was significantly positively associated with TI (β = 0.597, *p* < 0.001). The model explained 35.6% of the variance (R^2^ = 0.356, F = 533.507).

8. Association between SE and JSA

SE was significantly positively associated with JSA (β = 0.456, *p* < 0.001). The model explained 20.8% of the variance (R^2^ = 0.208, F = 253.621).

9. Association between SE and TI

SE was significantly negatively associated with TI (β = −0.249, *p* < 0.001). The model explained 6.2% of the variance (R^2^ = 0.062, F = 63.607).

10. Association between JSA and TI

JSA was significantly negatively associated with TI (β = −0.102, *p* < 0.001). The model explained 5.2% of the variance (R^2^ = 0.052, F = 52.595).

### 3.4. Path Analysis

This study employed path analysis to examine the relationships among MF, JS, SE, JSA, and TI. The structural framework was used to test whether the hypothesized relationships among these constructs were supported. The results are presented in Table 4.

#### 3.4.1. Path Analysis of MF, JS, SE, and JSA on TI

Model 1: This study first examined the direct associations of MF, JS, SE, and JSA with TI. The overall model explained 38.8% of the variance in TI (adjusted R^2^ = 0.385), indicating strong predictive power for understanding the turnover tendencies of direct care workers.

The results revealed a significant positive association between JS and TI (β = 0.599, *p* < 0.001), suggesting that higher levels of job stress are strongly linked to greater turnover intention. Conversely, JSA demonstrated a significant negative association with TI (β = −0.133, *p* < 0.001), indicating that higher job satisfaction substantially reduces employees’ intention to leave. In contrast, the direct associations of MF and SE with TI did not reach statistical significance (β = −0.035, *p* > 0.05; β = −0.046, *p* > 0.05). These findings imply that while these psychological resources may not directly predict turnover intention, they could still play a supportive role in shaping employees’ coping strategies and workplace attitudes.

A further simplified analysis (Model 1R), which excluded SE due to its nonsignificant contribution (*p* > 0.05), yielded consistent results. The revised model explained 38.1% of the variance in TI, with JS (β = 0.599, *p* < 0.001) and JSA (β = −0.139, *p* < 0.001) remaining the key associated factors. This result underscores a crucial pattern: higher job stress combined with lower job satisfaction significantly increases turnover intention among direct care workers. These empirical findings provide valuable implications for workforce management strategies and retention policies in long-term care settings.

The results of this study indicate that H2 (JS→TI) and H10 (JSA→TI) were supported, whereas H4 (MF→TI) and H7 (SE→TI) did not reach statistical significance in the path model, suggesting their influence is primarily indirect.

#### 3.4.2. Path Analysis of MF, JS, and SE on JSA

Model 2: We examined the direct effects of MF, JS, and SE on JSA. The overall model accounted for 27.6% of the variance in JSA (adjusted R^2^ = 0.274), indicating that these psychological and occupational variables provide meaningful explanatory power for understanding care workers’ job satisfaction. The results revealed that MF had a positive association on JSA (β = 0.187, *p* < 0.001), suggesting that a higher level of MF contributes to a stronger sense of satisfaction in the caregiving role. SE also exerted a positive association on JSA (β = 0.407, *p* < 0.001), demonstrating that individuals with greater confidence in their professional capabilities tend to experience higher satisfaction at work. Interestingly, JS exhibited a statistically significant positive association with JSA (β = 0.230, *p* < 0.001), contrary to the hypothesized negative relationship. This indicates that greater stress tends to be associated with higher levels of JSA, suggesting that care workers may interpret demanding or high-pressure situations as opportunities for accomplishment, thereby enhancing their job satisfaction.

The results of this study support H3 (MF→JSA) and H6 (SE→JSA), both demonstrating positive associations consistent with the theoretical expectations. However, H8 (JS→JSA) revealed a positive rather than the hypothesized negative association. This unexpected finding represents a key contribution of this study, indicating that job stress may function differently within care work contexts. Specifically, moderate or challenge-oriented stress may activate problem-solving motivation or generate a sense of achievement, which in turn enhances job satisfaction.

Overall, these results highlight the importance of MF and SE as psychological resources that promote JSA, while also suggesting that the role of JS may be more complex than traditionally assumed. This underscores the need to distinguish between harmful stress and challenge-based stress in long-term care workforce management and to develop strategies that help workers reframe demanding tasks as meaningful or growth-oriented experiences.

#### 3.4.3. Path Analysis of MF and JS on SE

Model 3: This model examined the effects of MF and JS on SE. The overall model explained 28.0% of the variance in SE (adjusted R^2^ = 0.279), indicating a satisfactory level of predictive power for caregivers’ SE. The results revealed a significant positive association between mindfulness and SE (β = 0.497, *p* < 0.001), suggesting that caregivers with higher MF tend to have stronger SE, enabling them to better handle workplace challenges and perform tasks effectively. Moreover, JS showed a significant negative association with SE (β = −0.109, *p* < 0.001), indicating that higher stress levels may undermine caregivers’ confidence in their abilities. The results of this study support H1 (MF→SE) and H5 (JS→SE). Overall, these findings suggest that enhancing mindfulness can strengthen self-efficacy, while reducing JS can further facilitate its development, both of which are crucial for improving job performance and workplace adaptability.

#### 3.4.4. Path Analysis of MF on JS

Model 4: This model examined the effect of MF on JS. The overall model explained 4.0% of the variance in JS (adjusted R^2^ = 0.039). Although the explanatory power was relatively modest, the results remained statistically significant. The analysis indicated a significant negative association between MF and JS (β = −0.200, *p* < 0.001). The results of this study support H2 (MF→JS), suggesting that caregivers with higher MF are better equipped to cope with workplace demands and experience reduced stress levels. These findings highlight the potential role of MF as a psychological resource for stress regulation and underscore its value as an effective strategy for enhancing resilience and reducing workplace stress.

**Figure 2:**
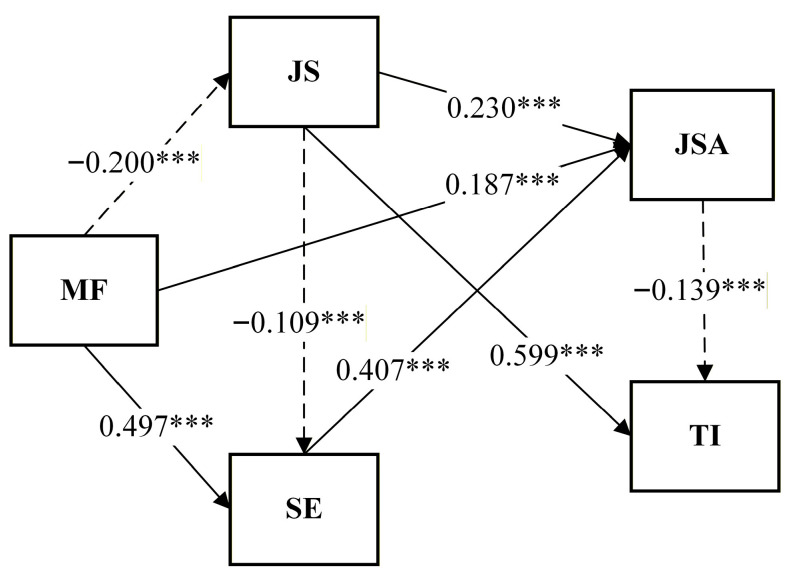
Results of the path analysis, *** *p* < 0.001.

The final results of the path analysis are presented in Figure 2, where solid lines indicate positive relationships between variables, and dashed lines denote negative relationships.

In summary, the path analysis reveals a comprehensive resource-driven mechanism. Rather than direct predictors, Mindfulness and Self-Efficacy function as foundational psychological resources that shape turnover intention through two primary indirect pathways: (1) by mitigating job stress and its subsequent health-impairment effects, and (2) by significantly augmenting job satisfaction, which serves as the most critical proximal buffer against withdrawal intentions. This broader pattern underscores that the retention of care workers depends less on isolated traits and more on the interplay between personal resources and intervening work perceptions (JS and JSA).

## 4. Discussion

This study elucidates the complex psychological mechanisms underlying turnover intention (TI) among direct care workers. By employing a comprehensive path model, this research delineates the sequential interplay between internal psychological resources (Mindfulness and Self-Efficacy) and external job perceptions (Job Stress and Job Satisfaction).

The findings indicate that Mindfulness (MF) is significantly negatively associated with Job Stress (JS) (β = −0.200, *p* < 0.001) while augmenting Self-Efficacy (SE) (β = 0.497, *p* < 0.001), providing empirical validation for the Conservation of Resources (COR) theory. In high-strain care environments, MF operates as a critical personal resource that buffers against resource depletion. Furthermore, the model reveals a paradoxical positive association between JS and Job Satisfaction (JSA) (β = 0.230, *p* < 0.001). This finding is consistent with recent empirical evidence reported by Jokanović et al. [59], who observed a positive association between job pressure and satisfaction when other stressors were statistically controlled. Similar conclusions were drawn by Singh et al. [60], suggesting that workload does not inevitably result in psychological strain.

This relationship can be theoretically interpreted through the Challenge–Hindrance Stressor Framework. As proposed by Webster et al. [61], challenge demands may stimulate motivation and achievement rather than exhaustion. Additionally, Khatun et al. [62] noted that work stress, up to a certain threshold, can function as a motivational force. For caregivers with high SE and MF, job demands are cognitively reappraised as “challenge stressors” that promote professional mastery.

A pivotal observation is the attenuation of SE’s direct effect on TI (β = −0.046, *p* > 0.05) within the comprehensive path model. This finding implies a hierarchical influence structure, where SE primarily fosters JSA—the most potent predictor of TI—to indirectly suppress withdrawal intentions. This aligns with the Job Demands–Resources (JD-R) model’s motivational process, emphasizing that personal resources shape the appraisal of demands. Furthermore, multicollinearity diagnostics (VIF < 1.63) suggest that these relationships are statistically robust.

Positive psychological traits serve as distal personal resources that support functioning in long-term care; however, their influence on turnover intention is not direct but operates entirely through proximal work-related experiences, such as job stress and satisfaction [63].

### 4.1. Limitations and Future Research

Despite its theoretical contributions, this study has several limitations that warrant consideration. First, the cross-sectional design inherently restricts the inference of temporal causality among mindfulness, stress, and turnover intention. Although the low VIF diagnostics and Harman’s single-factor test suggest that common method bias (CMB) and multicollinearity do not appear to pose serious concerns, future research should employ longitudinal designs or multiple data sources (e.g., supervisor evaluations) to strengthen causal claims and mitigate same-source bias, such as through marker-variable techniques.

Second, to maintain model parsimony, this research primarily focused on internal psychological resources. The influence of these resources may be relatively modest compared to structural or organizational determinants (e.g., compensation or administrative support), which were not the primary focus of this model. This exclusion may limit the model’s contextual explanatory power. While procedural controls were implemented, the absence of latent variable modeling (SEM) and bootstrapped indirect effects should be acknowledged as a methodological constraint. This may limit the ability to fully account for measurement errors and complex mediation nuances compared to more sophisticated structural models. Future studies should utilize multilevel analysis (HLM) or multigroup SEM as well as investigate specific dimensions of job stress (e.g., workload vs. role conflict), to disentangle these nuanced effects and further validate the positive JS–JSA association”.

Finally, the use of convenience sampling and the specific demographic concentration (e.g., the 50–59 age group) may affect the generalizability of the findings. Future research should transition to latent variable modeling and employ stratified sampling to enhance external validity across diverse cultural and professional landscapes. Exploring how variations in income levels and specific workload characteristics moderate the resource–retention link will further deepen the practical implications for the global long-term care industry.

### 4.2. Practical Implications

Promote MF Training and Psychological Support Programs: Given the significant role of MF in reducing job stress and enhancing SE, it is recommended that organizations integrate MF training into employee development programs. This can not only improve employees’ mental health but also enhance their job performance and satisfaction, thereby reducing TI. Furthermore, employees should be encouraged to adopt MF techniques to cope with daily challenges at work, thereby boosting their psychological resilience.

Optimize JS Management Strategies: The study shows that job stress is a key factor influencing TI. Organizations should aim to mitigate employee job stress. Specific measures include improving workload distribution, creating flexible work environments, and providing psychological health support for employees. Additionally, organizations should regularly assess JS levels and adjust work processes and support mechanisms based on employee needs. To ensure these measures are effective, organizations must prioritize structural reforms such as enhancing role clarity and strengthening administrative support systems. For instance, implementing peer-support groups can help mitigate the direct impact of job demands, thereby fostering a supportive environment that complements individual-level training.

Enhance Employee SE: SE has a profound impact on employees’ JSA and TI. Therefore, organizations should design targeted training and development plans to help employees enhance their SE. To effectively mitigate turnover intention, healthcare organizations should prioritize resource-based interventions that enhance employees’ Job Satisfaction and Achievement (JSA). Such interventions should include skills enhancement programs and psychological or emotional management support, which empower employees by strengthening their sense of professional control and competence. In addition, organizations are encouraged to implement systematic organizational measures—such as optimizing compensation structures and fostering a supportive and respectful work environment—to further promote JSA. As JSA functions as a critical buffer against turnover intention, strengthening these factors should be regarded as a strategic managerial priority to ensure the long-term sustainability of the long-term care workforce, particularly in the context of an aging society.

## 5. Conclusions

### Practical Recommendations

This study reaches a clear conclusion that MF and SE do not directly reduce TI, but instead influence TI through indirect pathways associated with employees’ work experiences. In particular, job satisfaction and achievement (JSA) and JS function as the key mechanisms through which these psychological resources exert their effects.

This study highlights the critical role of MF and SE as essential personal resources for direct care workers in Taiwan’s super-aged society. The findings demonstrate that while these psychological traits do not directly reduce TI, they function through indirect pathways by significantly enhancing JSA and mitigating the negative impacts of JS. By clarifying these resource-driven mechanisms within the JD-R and COR frameworks, this research provides a scientific foundation for sustaining the long-term care workforce as demographic demands intensify.

## Figures and Tables

**Table 1:** Summary of Reliability and Factor Analysis for Each Construct.

Construct	α	KMO Value	Bartlett’s Test of Sphericity			Cumulative Explained Variance (%)	Factor Loadings
			Approx. Chi-Square	df	Sig		
MF	0.819	0.864	5853.155	91	0.000	62.650	0.649–0.867
JS	0.882	0.941	21,152.737	378	0.000	71.118	0.705–0.881
SE	0.775	0.863	4930.1276	55	0.000	61.143	0.536–0.868
JSA	0.866	0.927	10,561.838	105	0.000	66.245	0.676–0.889
TI	0.958	0.952	11,543.133	66	0.000	69.830	0.580–0.891

**Table 2:** Demographic Characteristics of Respondents (*n* = 967).

Variable	*n*	(%)
Gender		
Male	148	15.3
Female	819	84.7
Marital Status		
Single	428	44.3
Married	539	55.7
Nationality		
Republic of China (Taiwan)	868	89.8
Other Countries	99	10.2
Age		
20–29 years	85	8.8
30–39 years	144	14.9
40–49 years	254	26.3
50–59 years	311	32.2
60 years and above	173	17.9
Education Level		
Junior high school or below	136	14.1
Senior high school (vocational)	457	47.3
College or university	366	37.8
Graduate school or above	8	0.8
Total Years of Experience as a Caregiver		
Less than 1 year	136	14.1
1 to 3 years (inclusive)	262	27.1
3 to 5 years (inclusive)	168	17.4
5 to 7 years (inclusive)	148	15.3
7 to 9 years (inclusive)	68	7.0
9 to 11 years (inclusive)	57	5.9
More than 11 years	128	13.2
Number of Clients Cared for per Week		
1–2 clients	60	6.2
3–4 clients	210	21.7
5–6 clients	395	40.8
7 or more clients	302	31.2
Monthly Income (NTD)		
20,000–35,000	464	48.0
35,001–50,000	346	35.8
50,001–65,000	111	11.5
65,001–80,000	31	3.2
80,001–95,000	9	0.9
More than 95,000	6	0.6

**Table 3:** Summary of Regression Analysis Results.

Path	β	R^2^	Adj. R^2^	F
MF→JS	−0.200 ***	0.040	0.039	40.356
MF→SE	0.518 ***	0.269	0.268	354.722
MF→JSA	0.352 ***	0.124	0.123	136.813
MF→TI	−0.225 ***	0.051	0.050	51.391
JS→SE	−0.209 ***	0.044	0.043	44.209
JS→JSA	0.107 ***	0.011	0.010	11.222
JS→TI	0.597 ***	0.356	0.355	533.507
SE→JSA	0.456 ***	0.208	0.207	253.621
SE→TI	−0.249 ***	0.062	0.061	63.607
JSA→TI	−0.102 ***	0.011	0.009	10.243

Note: *** *p* < 0.001.

**Table 4:** Summary of Multiple Regression Analysis.

Construct	Model 1	Model 1R	Model 2	Model 3	Model 4
	TI	TI	JSA	SE	JS
MF	−0.035	×	0.187 ***	0.497 ***	−0.200 ***
JS	0.599 ***	0.599 ***	0.230 ***	−0.109 ***	×
SE	−0.046	×	0.407 ***	×	×
JSA	−0.133 ***	−0.139 ***	×	×	×
R^2^	0.388	0.381	0.276	0.280	0.040
Adjusted R^2^	0.385	0.385	0.274	0.279	0.039
F Value	152.172	202.634	122.595	187.701	40.356
VIF Values	1.131–1.618	1.105–1.335	1.058–1.389	1.042	1.000

Notes: *** *p* < 0.001, “×” indicates that the variable was not included in the specific model analysis.

## Data Availability

The data presented in this study are available on request from the corresponding author due to ethical restrictions and participant privacy concerns.

## References

[1] National Development Council (2024). Population Projections for the R.O.C. (Taiwan): 2024–2070.

[2] Jurij R., Ismail I.R., Alavi K., Alavi R. (2023). Eldercare’s Turnover Intention and Human Resource Approach: A Systematic Review. Int. J. Environ. Res. Public Health.

[3] Ning L., Jia H., Gao S., Liu M., Xu J., Ge S., Li M., Yu X. (2023). The Mediating Role of Job Satisfaction and Presenteeism on the Relationship between Job Stress and Turnover Intention among Primary Health Care Workers. Int. J. Equity Health.

[4] Alzoubi M.M., Al-Mugheed K., Oweidat I., Alrahbeni T., Alnaeem M.M., Alabdullah A.A.S., Abdelaliem S.M.F., Hendy A. (2024). Moderating Role of Relationships between Workloads, Job Burnout, Turnover Intention, and Healthcare Quality among Nurses. BMC Psychol..

[5] Chaturvedi A. (2024). Impact of Job Satisfaction on Employee Turnover Intent. SSRN.

[6] Dodanwala T.C., Santoso D.S. (2022). The Mediating Role of Job Stress on the Relationship between Job Satisfaction Facets and Turnover Intention of the Construction Professionals. Eng. Constr. Archit. Manag..

[7] Kim H.-K., Seo J.-H., Park C.-H. (2022). The Mediating Effect of Self-Efficacy and Coping Strategy in Relation to Job Stress and Psychological Well-Being of Home-Visiting Care Workers for Elderly during the COVID-19 Pandemic. Int. J. Environ. Res. Public Health.

[8] Yuan D., Hu M., Yao N., Zhong H., Xiao Y., Zhou X., Zhang R., Zhang Y. (2024). Effects of Perceived Stress on Turnover Intention of Female Healthcare Staff: A Serial Multiple Mediation Model. BMC Public Health.

[9] Abudaqa A., Hilmi M.F., Dahalan N. (2022). The Nexus between Job Burnout and Emotional Intelligence on Turnover Intention in Oil and Gas Companies in the UAE. arXiv.

[10] Alkhraish M.Y., Eivazzadeh N., Yeşiltaş M. (2023). The Impact of Burnout on Turnover Intention Among Nurses: The Mediating Role of Job Satisfaction. Hacet. Sağlık İdaresi Derg..

[11] Faridah F., Gustini G., Salehan S., Efendi R. (2022). The Turnover Intention Influenced by Job Satisfaction and Organizational Commitment. Int. J. Soc. Sci. Res. Rev..

[12] Kabat-Zinn J. (1994). Wherever You Go, There You Are: Mindfulness Meditation in Everyday Life.

[13] Charoensukmongkol P., Puyod J.V. (2022). Mindfulness and Emotional Exhaustion in Call Center Agents in the Philippines: Moderating Roles of Work and Personal Characteristics. J. Gen. Psychol..

[14] Coo C., Salanova M. (2018). Mindfulness Can Make You Happy-and-Productive: A Mindfulness Controlled Trial and Its Effects on Happiness, Work Engagement and Performance. J. Happiness Stud..

[15] Vonderlin R., Biermann M., Bohus M., Lyssenko L. (2020). Mindfulness-Based Programs in the Workplace: A Meta-Analysis of Randomized Controlled Trials. Mindfulness.

[16] Kumprang K., Suriyankietkaew S. (2024). Mechanisms of Organizational Mindfulness on Employee Well-Being and Engagement: A Multi-Level Analysis. Adm. Sci..

[17] Shrestha S., Alharbi R.J.M., While C., Ellis J., Rahman M.A., Wells Y. (2021). Self-Efficacy of Direct Care Workers Providing Care to Older People in Residential Aged Care Settings: A Scoping Review Protocol. Syst. Rev..

[18] Jia H., Gao S., Shang P., Cao P., Yu J., Yu X. (2022). The Relationship between Public Service Motivation and Turnover Intention: The Mediating Role of Work Stress and Task Performance. Environ. Health Prev. Med..

[19] Bakker A.B., Demerouti E. (2017). Job Demands–Resources Theory: Taking Stock and Looking Forward. J. Occup. Health Psychol..

[20] Demerouti E., Bakker A.B., Nachreiner F., Schaufeli W.B. (2001). The Job Demands–Resources Model of Burnout. J. Appl. Psychol..

[21] Hobfoll S.E. (2011). Conservation of Resources Theory: Its Promise and Prospects.

[22] Malinowski P., Lim H.J. (2015). Mindfulness at Work: Positive Affect, Hope, and Optimism Mediate the Relationship Between Dispositional Mindfulness, Work Engagement, and Well-Being. Mindfulness.

[23] Grover S.L., Teo S.T.T., Pick D., Roche M. (2017). Mindfulness as a Personal Resource to Reduce Work Stress in the Job Demands-resources Model. Stress Health.

[24] Reitz M., Waller L., Chaskalson M., Olivier S., Rupprecht S. (2020). Developing Leaders through Mindfulness Practice. J. Manag. Dev..

[25] Di Gesto C., Policardo G.R., Benucci S.B., Çela E., Grano C. (2025). Difficulties in Emotion Regulation and Stress in Intensive Care Unit Nurses During COVID-19: Exploring the Mediating Role of Psychological Inflexibility and the Moderating Effect of Work Experience. Healthcare.

[26] Chami-Malaeb R. (2022). Relationship of Perceived Supervisor Support, Self-Efficacy and Turnover Intention, the Mediating Role of Burnout. Pers. Rev..

[27] Kurniawan M.H., Hariyati R.T.S., Afifah E. (2019). The Relationship between Caring Preceptor, Self-Efficacy, Job Satisfaction, and New Nurse Performance. Enferm. Clínica.

[28] Kim H., Park D. (2023). Effects of Nursing Professionalism and Self-Efficacy on Job Embeddedness in Nurses. Heliyon.

[29] Rafiei S., Souri S., Nejatifar Z., Amerzadeh M. (2024). The Moderating Role of Self-Efficacy in the Relationship between Occupational Stress and Mental Health Issues among Nurses. Sci. Rep..

[30] Bernales-Turpo D., Quispe-Velasquez R., Flores-Ticona D., Saintila J., Ruiz Mamani P.G., Huancahuire-Vega S., Morales-García M., Morales-García W.C. (2022). Burnout, Professional Self-Efficacy, and Life Satisfaction as Predictors of Job Performance in Health Care Workers: The Mediating Role of Work Engagement. J. Prim. Care Community Health.

[31] Chuang C.-H., Tseng P.-C., Lin C.-Y., Lin K.-H., Chen Y.-Y. (2016). Burnout in the Intensive Care Unit Professionals: A Systematic Review. Medicine.

[32] Imran B., Mariam S., Aryani F., Ramli A.H. (2020). Job Stress, Job Satisfaction and Turnover Intention. Proceedings of the International Conference on Management, Accounting, and Economy (ICMAE 2020).

[33] Tran C.T.H., Tran H.T.M., Nguyen H.T.N., Mach D.N., Phan H.S.P., Mujtaba B.G. (2020). Stress Management in the Modern Workplace and the Role of Human Resource Professionals. Bus. Ethics Leadersh..

[34] Al Sabei S.D., Labrague L.J., Miner Ross A., Karkada S., Albashayreh A., Al Masroori F., Al Hashmi N. (2020). Nursing Work Environment, Turnover Intention, Job Burnout, and Quality of Care: The Moderating Role of Job Satisfaction. J. Nurs. Scholarsh..

[35] Ali B.J., Anwar G. (2021). Employee Turnover Intention and Job Satisfaction. Int. J. Adv. Eng. Manag. Sci..

[36] Chapagain R. (2020). Employee Turnover Intention in a Service Industry: A Systematic Literature Review. Migr. Lett..

[37] Hu H., Wang C., Lan Y., Wu X. (2022). Nurses’ Turnover Intention, Hope and Career Identity: The Mediating Role of Job Satisfaction. BMC Nurs..

[38] Ganji S.F.G., Johnson L.W., Sorkhan V.B. (2021). The Effect of Employee Empowerment, Organizational Support, and Ethical Climate on Turnover Intention: The Mediating Role of Job Satisfaction. Iran. J. Manag. Stud..

[39] Mirzaei A., Imashi R., Saghezchi R.Y., Jafari M.J., Nemati-Vakilabad R. (2024). The Relationship of Perceived Nurse Manager Competence with Job Satisfaction and Turnover Intention among Clinical Nurses: An Analytical Cross-Sectional Study. BMC Nurs..

[40] Liu Y., Duan Y., Guo M. (2023). Turnover Intention and Its Associated Factors among Nurses: A Multi-Center Cross-Sectional Study. Front. Public Health.

[41] Xu K., Lei L., Guo Z., Liu X., Shi Y., Han G., Lin K., Cai W., Lu C., Li X. (2024). Turnover Intention among Healthcare Workers in Shenzhen, China: The Mediating Effect of Job Satisfaction and Work Engagement. BMC Health Serv. Res..

[42] Cohen J. (1988). Statistical Power Analysis for the Behavioral Sciences.

[43] Brown K.W., Ryan R.M. (2003). The Benefits of Being Present: Mindfulness and Its Role in Psychological Well-Being. J. Pers. Soc. Psychol..

[44] Baer R.A., Smith G.T., Hopkins J., Krietemeyer J., Toney L. (2006). Using Self-Report Assessment Methods to Explore Facets of Mindfulness. Assessment.

[45] Zivnuska S., Kacmar K.M., Ferguson M., Carlson D.S. (2016). Mindfulness at Work: Resource Accumulation, Well-Being, and Attitudes. Career Dev. Int..

[46] Caplan R.D., Cobb S., French J.R.P., Van Harrison R., Pinneau S.R. (1975). Job Demands and Worker Health: Main Effects and Occupational Differences.

[47] Parker D.F., DeCotiis T.A. (1983). Organizational Determinants of Job Stress. Organ. Behav. Hum. Perform..

[48] Zhang J.X., Schwarzer R. (1995). Measuring Optimistic Self-Beliefs: A Chinese Adaptation of the General Self-Efficacy Scale. Psychol. Int. J. Psychol. Orient.

[49] Bandura A. (1986). Social Foundations of Thought and Action: A Social Cognitive Theory.

[50] Riggs M.L., Warka J., Babasa B., Betancourt R., Hooker S. (1994). Development and Validation of Self-Efficacy and Outcome Expectancy Scales for Job-Related Applications. Educ. Psychol. Meas..

[51] Weiss D.J., Dawis R.V., England G.W., Lofquist L.H. (1967). Manual for the Minnesota Satisfaction Questionnaire.

[52] Babin B.J., Boles J.S. (1998). Employee Behavior in a Service Environment: A Model and Test of Potential Differences between Men and Women. J. Mark..

[53] Cammann C., Fichman M., Jenkins G.D., Klesh J.R., Seashore S., Lawler E., Mirvis P., Cammann C. (1983). Assessing the Attitudes and Perceptions of Organizational Members. Assessing Organizational Change: A Guide to Methods, Measures, and Practices.

[54] Scott C.R., Connaughton S.L., Diaz-Saenz H.R., Maguire K., Ramirez R., Richardson B., Shaw S.P., Morgan D. (1999). The Impacts of Communication and Multiple Identifications on Intent to Leave: A Multimethodological Exploration. Manag. Commun. Q..

[55] Jung H.S., Namkung Y., Yoon H.H. (2010). The Effects of Employees’ Business Ethical Value on Person–Organization Fit and Turnover Intent in the Foodservice Industry. Int. J. Hosp. Manag..

[56] Kelloway E.K., Gottlieb B.H., Barham L. (1999). The Source, Nature, and Direction of Work and Family Conflict: A Longitudinal Investigation. J. Occup. Health Psychol..

[57] Mobley W.H., Horner S.O., Hollingsworth A.T. (1978). An Evaluation of Precursors of Hospital Employee Turnover. J. Appl. Psychol..

[58] Michaels C.E., Spector P.E. (1983). “Causes of employee turnover: A test of the Mobley, Griffeth, Hand, and Meglino Model”: Correction to Michaels and Spector. J. Appl. Psychol..

[59] Jokanović B., Vrgović P., Ćulibrk J., Tomić I., Jošanov-Vrgović I. (2025). Job Satisfaction in the Face of Organizational Stress: Validating a Stress Symptoms Survey and Exploring Stress-Related Predictors. Sustainability.

[60] Singh P., Bhardwaj P., Sharma S.K., Mishra V. (2022). Effect of Organizational Factors on Psychological Stress and Job Satisfaction. Vis. J. Bus. Perspect..

[61] Webster J.R., Beehr T.A., Christiansen N.D. (2010). Toward a Better Understanding of the Effects of Hindrance and Challenge Stressors on Work Behavior. J. Vocat. Behav..

[62] Khatun A., Bharti V., Tiwari M. (2022). Effects of Work Stress on Psychological Well-Being and Job Satisfaction: A Review. Revisioning and Reconstructing Paradigms and Advances in Industry.

[63] Amah O.E. (2014). Challenge and Hindrance Stress Relationship with Job Satisfaction and Life Satisfaction: The Role of Motivation-to-Work and Self-Efficacy. Int. J. Humanit. Soc. Sci..

